# The sympathetic nervous response in inflammation

**DOI:** 10.1186/s13075-014-0504-2

**Published:** 2014-12-12

**Authors:** Georg Pongratz, Rainer H Straub

**Affiliations:** Department of Internal Medicine I, University Hospital, Laboratory of Experimental Rheumatology and Neuroendocrine Immunology, 93042 Regensburg, Germany

## Abstract

Over the past decades evidence has accumulated clearly demonstrating a pivotal role for the sympathetic nervous system (SNS) and its neurotransmitters in regulating inflammation. The first part of this review provides the reader with an overview showing that the interaction of the SNS with the immune system to control inflammation is strongly context-dependent (for example, depending on the activation state of the immune cell or neuro-transmitter concentration). In the second part we focus on autoimmune arthritis as a well investigated example for sympathetically controlled inflammation to show that the SNS and catecholamines play a differential role depending on the time point of ongoing disease. A model will be developed to explain the proinflammatory effects of the SNS in the early phase and the anti-inflammatory effects of catecholamines in the later phase of autoimmune arthritis. In the final part, a conceptual framework is discussed that shows that a major purpose of increased SNS activity is nourishment of a continuously activated immune system at a systemic level using energy-rich fuels (glucose, amino acids, lipids), while uncoupling from central nervous regulation occurs at sites of inflammation by repulsion of sympathetic fibers and local adrenoceptor regulation. This creates zones of ‘permitted local inflammation’. However, if this ‘inflammatory configuration’ persists and is strong, as in autoimmunity, the effects are detrimental because of the resultant chronic catabolic state, leading to cachexia, high blood pressure, insulin resistance, and increased cardiovascular mortality, and so on. Today, the challenge is to translate this conceptual knowledge into clinical benefit.

## Introduction

The sympathetic nervous system (SNS) is an integrative system that reacts to dangerous situations, and activation of the SNS is part of the classical ‘fight and flight’ response. This is common knowledge. However, the SNS is not active just in these extreme situations, but is part of constant regulatory machinery that keeps body functions in a steady-state equilibrium. Of course, the SNS is not alone in performing these tasks but is interwoven into complex regulatory circuits. Therefore, it is not possible to analyze the action of the SNS in inflammation without considering the other important players, like the hypothalamic-pituitary-adrenal (HPA) axis, and the sensory nervous system and vagal nervous system (VNS). For a detailed description of the functional anatomy of the autonomous (SNS and VNS) and sensory nervous system, as well as the HPA axis, we refer the reader to respective standard textbooks of physiology since this is established and common knowledge and a detailed description would go beyond the scope of this review. In the first part of this review we focus on important highlights concerning the SNS and inflammation. In the second part, the standalone facts will be integrated to try to understand the deeper meaning of this regulatory machinery in inflammatory disease. As an example, we refer to findings concerning neuroendocrine immune regulation in arthritis.

## Review criteria

This review is based on a systematic search of the PubMed database using the search terms ‘sympathetic nervous system’, ‘peripheral nervous system’, ‘nerve fiber’, ‘neuroimmun*’, ‘norepinephrine’, ‘arthritis’, ‘collagen induced arthritis’, ‘rheumatoid arthritis’, ‘autoimmune diseases’, ‘autoimmunity’. Articles (including abstracts) published in English or German up to March 2014 were considered. All retrieved articles were screened for eligibility based on title, abstract, and full content.

## The sympathetic nervous system and inflammation

It was noted some time ago that the SNS and inflammation are close partners. One of the first mentions of the influence of the SNS on inflammation can be found in an article from 1903. The authors performed surgical local sympathectomy of the ear of rabbits after provoking inflammation by inoculation with staphylococci. They concluded that ‘….relations of the sympathetic nerve … to the course of inflammation, … are due to some nervous functions of the sympathetic nerve other than… vasoconstriction and vasodilatation’ [1]. Already in 1936, Reilly speculated that endotoxin concentrates in sympathetic tissue and irritates sympathetic nerve fibers, which results in a systemic reaction that resembles symptoms of typhoid fever [2]. This view, of course, was very rudimentary but this theory already implied that there is some crosstalk between the SNS and inflammation, and that both systems interact with each other.

Today our understanding of this relationship is more detailed. When an antigen enters the body, local activation of immune cells leads to release of proinflammatory mediators, which are able to excite or lower thresholds of afferent nociceptive and afferent vagal nerve fibers [3]. If the neuronal signal strength is strong enough or if spillover of local inflammatory mediators into the circulation is robust enough, it signals to the brain, resulting in activation of the two major stress axes, the HPA axis and the SNS [3,4]. Cytokines like interleukin (IL)-1β [3,5] or tumor necrosis factor (TNF) [6] produced by locally activated innate immune cells are pivotal in this communication from immune system to central nervous system.

*Vice versa*, central sympathetic activity has a direct impact on inflammatory cytokines. In a study with hypertensive patients, central inhibition of the SNS decreased peripheral TNF serum levels [7]. In another study, sympathetic tone was positively correlated with IL-6 plasma levels [8]. Similarly, stress responses that modulate SNS activity have great impact on inflammation [9]. However, there might be a disruption of this communication between the brain and the immune system in the course of protracted inflammation, as shown in an arthritis model in rats [10]. This disruption is beneficial on a systemic level, which is discussed below.

In the mid-1980s, it was recognized that secondary lymphoid tissue is highly innervated by sympathetic nerve fibers and sympathetic nerve terminals are found in close proximity to immune cells, especially in primary and secondary lymphoid tissue [11]. Immune cells express receptors for neurotransmitters, for example, adrenoceptors (ARs), which are functional and translate neuronal signals into immune cell signals [12]. The communication between brain and inflamed area can be disturbed, for example, by a stroke, which results in asymmetric inflammation. This can lead to reduced inflammation on the paralyzed side in rheumatoid arthritis, which was already recognized in 1962 [13].

In this respect, it has been shown that patients with minor stroke [14] or poliomyelitis [15] show weaker delayed type hypersensitivity (DTH) responses on the paretic side. After excluding changes in blood flow, the authors of the latter study concluded that ‘… another mechanism, such as a direct effect of sympathetic transmitters on inflammatory cells, may mediate the putative effects of the SNS on DTH responses.’

Another clinically well recognized phenomenon after stroke is immunosuppression. In a rat model of stroke, the authors observed reduced infection rates following sympathectomy, indicating SNS-mediated immunosuppression [16], which might depend on the type of infectious agent [17].

The activation of the SNS in the context of an active immune system results in release of sympathetic neurotransmitters. Notably, sympathetic nerves release not only norepinephrine (NE) as the major neurotransmitter, but also ATP, neuropeptide Y (NPY), and nitric oxide [18]. All neurotransmitters have direct influence on immune cells, although NE is the best characterized in this respect. NPY, for example, has been shown to increase adhesion of human leukocytes to endothelial cells [19], and the NPY antagonist PP56 showed anti-inflammatory effects in acute carrageenan-induced arthritis and chronic adjuvant arthritis [20].

**Figure 1:**
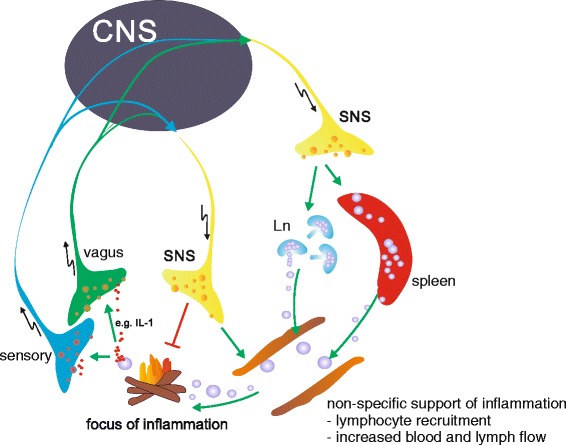
**Basic neuronal anti-inflammatory reflex.** Local inflammation (the fire) is detected by vagal and sensory nerve fibers, which express receptors for inflammatory mediators, like interleukin (IL)-1β (red dots). An afferent signal is generated and transmitted to the brain (central nervous sytem (CNS)), which in turn leads to activation of the sympathetic nervous system (SNS), which has a complex impact on inflammation. Local release of SNS neurotransmitters, like norepinephrine, at the site of inflammation or in secondary lymphoid organs has a net anti-inflammatory outcome. On the other hand, non-specific immune stimulatory processes on a systemic level are supported, like recruitment of leukocytes, increased blood and lymph flow, but also increasing antigen processing and presentation and provision of energy-rich fuels. Ln, lymph node.

Sympathetic influence on immune cells can be direct, via ARs on immune cells [4], or indirect via regulating blood or lymph flow [21], regulating distribution [22] and production [23] of lymphocytes, or modulating the release of proinflammatory peptides [24], like substance P from sensory nerve endings, which among others express α-ARs [25] (Figure 1). Inflammatory cell recruitment and redistribution is also controlled by the SNS (Figure 1). One study showed that regulation of circadian changes in leukocyte distribution involves, among others, the activity of the SNS via β-ARs expressed on non-hematopoietic cells, leading to tissue-specific, differential circadian oscillations in the expression of endothelial cell adhesion molecules and chemokines [22]. Another study pointed out the role of SNS-dependent monocyte recruitment from the spleen in experimental peritoneal infection [17,26]. In addition, the generation of some leukocytes in the bone marrow is influenced by the SNS via β-ARs, resulting in preferential production of proinflammatory leukocyte populations [23].

As a side note, there is a direct interrelation between the SNS and the sensory nervous system, since the sensory response is significantly modulated by sympathetic signaling (for example, [27]). Such findings have also been discussed in the context of understanding clinical entities like the complex regional pain syndrome (for example, [28]).

TNF was the first cytokine whose production was shown to be regulated by occupation of α-ARs or β-ARs by catecholamines [29,30]. Subsequently, a whole array of other cytokines and immune cells has been demonstrated to be influenced by AR stimulation, both *in vitro* and *in vivo* (for example, [31]). Selected examples of the direct modulation of immune cell function by sympathetic neurotransmitters are presented in Table 1.

**Table 1:** **Examples of direct sympathetic neurotransmitter immune cell interactions**

**Cell type**	**Source**	**Receptor/neurotransmitter concentration**	**Costimulus**	**Main effect**	**Reference**
Macrophage	Bone marrow, mouse	NE 10^−6^ M	LPS	Decrease CCR2	[32]
		NE 10^−8^ M, 10^−6^ M	None	Decrease BMM proliferation	
		NE 10^−8^ M	LPS	Increase maturation	
		NE 10^−8^ M, 10^−6^ M	None	Increase phagocytosis	
		NE 10^−8^ M, 10^−6^ M	LPS	Increase TNF	
	Spleen, mouse	NE 10^−8^ M	LPS	Increase TNF	[29,33]
	Spleen, mouse	NE 10^−6^ M	LPS	Decrease TNF	[29,33]
	Peritoneum, mouse	Neuropeptide Y		Increase HMGB1	[34]
Dendritic cell	Bone marrow derived, mouse	β2-AR	NOD2 agonist	Increase IL6	[35]
			TLR2 agonist	Decrease IL-12	
		β2-AR		Increase IL-33	[36]
		α2-AR		Increase in antigen uptake	[37]
	Human cord blood	NE 10^−6^ M	LPS	Decrease IL12p40, TNF, IL-6, IL-23	[38]
T cell	Spleen, mouse	β2-AR	None	Increase Treg apoptosis	[39]
		β2-AR	Anti-CD3	Decrease IL-2 in CD4 + CD62L+ cells	[40]
		β2-AR	None	Increase in Treg mediated cell suppression	
	CIA, mouse	NE	ConA	Increase IFN-γ	[41]
	Splenic naïve T cells	β2-AR	Anti-CD3	Decrease IL-2	[42]
			Anti-CD28		
		β2-AR	Anti-CD3	Increase IFN-γ per Th1 cell	[43]
			Anti-CD28		
			IL-12		
	T cell clone	Neuropeptide Y	None	Decrease IFN-γ	[44]
				Increase IL-4	
B cell	CIA, mouse	β2-AR	Anti-CD40/IL-4	Increase IL-10	[45]
		β2-AR	Anti-CD40/IL-4	Inhibits IL-7 receptor signaling	[46]
	Naïve, splenic B cells	β2-AR	CD40L/IL-4	Increase IgG1, IgE	[47,48]

AR, adrenoceptor; BMM, bone marrow-derived macrophages; CCR2, C-C chemokine receptor type 2; CIA, collagen-induced arthritis; ConA, Concanavalin A; HMGB1, high-mobility-group-protein B1; IFN, interferon; Ig, immunoglobulin; IL, interleukin; LPS, lipopolysaccharide; NE, norepinephrine; NO2, nucleotide-binding oligomerization domain-containing protein 2; Th1, T helper 1; TLR2, toll-like receptor 2; TNF, tumor necrosis factor; Treg, regulatory T cell.

Also, pathogens use the sympathetic machinery to their advantage. For example, the cytomegalovirus immediate/early promotor can be stimulated directly via β2-ARs of monocytes, leading to reactivation of the virus [49]. NE release from sympathetic nerves in the gut is inhibited by infection with *Trichinella spiralis* to dampen the immune response against the pathogen [50].

**Figure 2:**
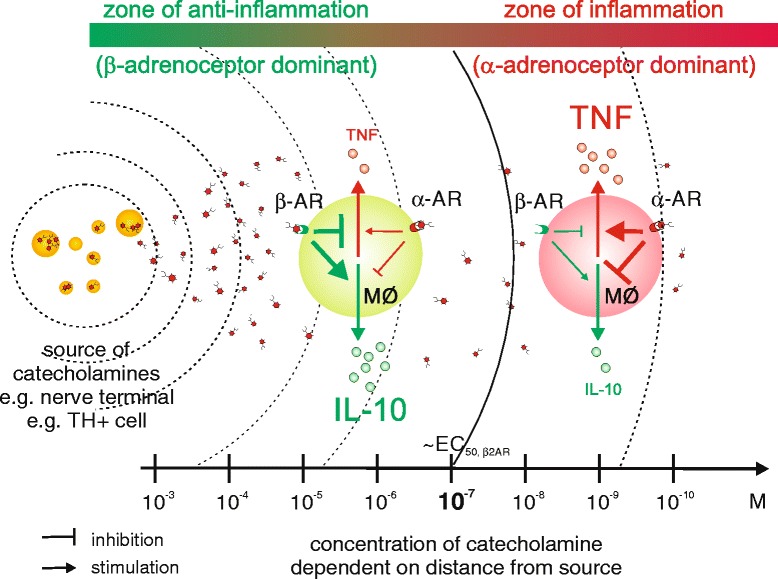
**Catecholamine effects depend on the distance from catecholamine source.** α- and β-adrenoceptors (ARs) show different binding affinities for catecholamines. Norepinephrine, the main neurotransmitter in the sympathetic nervous system (SNS), binds with higher affinity to α-ARs than β-ARs. Simultaneous expression of these receptors on immune cells (for example, macrophages (MΦ)) provides these cells with a passive means to determine the distance to the next catecholamine source. In close proximity to the catecholamine source (for example, sympathetic nerve terminal or catecholamine-producing tyrosine hydroxylase (TH)-positive cell) the concentration is high enough to activate β-ARs, whereas at a greater distance only α-ARs are activated. In the case of innate immune cells, like macrophages, this directly translates into anti-inflammatory (for example, increases in interleukin (IL)-10 via β-AR) or proinflammatory activity (for example, increases in tumor necrosis factor (TNF) via α-AR). Therefore, the simultaneous expression of α-ARs and β-ARs on immune cells provides a mean to regulate inflammatory processes dependent on the distance to the catecholamine source. We hypothesize that the body uses this system to promote local inflammation by repulsion of sympathetic nerve fibers from inflamed areas (zone of inflammation) and, at the same time, locally confines the inflammatory process by suppression of bystander activation in the zone of anti-inflammation.

The net effect of stimulating ARs on immune cells is not straightforward because it strongly depends on the context of exposure of receptive cells to sympathetic neurotransmitters; for example, the activation state of the cell [45,51], the proximity of the cell to the source of neurotransmitters (since this determines neurotransmitter concentration at the receptor; Figure 2), the presence of factors that modulate the adrenergic response [52], the pattern of AR expression on immune cells [53], or simply age [54].

Increasing the complexity of this matter, the VNS also has profound effects on inflammatory responses. The activity of the VNS is increased following endotoxemia. In this respect, an ‘anti-inflammatory reflex’ has been postulated, with the efferent vagus nerve acting in an anti-inflammatory manner via release of acetylcholine and activation of α7-nicotinic acetylcholine (nACh) receptors expressed on immune cells [55]. Since the spleen has no parasympathetic innervation, it has been hypothesized that the efferent part of the vagus activates splenic SNS fibers that release NE from SNS nerve endings in close proximity to immune cells. Upon stimulation of ARs on a subset of CD4 T cells, these cells release acetylcholine, which in turn has an immunosuppressive effect via α7-nACh receptors on macrophages [55]. However, this view has been challenged recently, since it has been shown by retrograde and anterograde staining and electrophysiological experiments that there is no neural connection from VNS to SNS projecting to the spleen [56]. This challenges the view that the vagus is indeed the effector arm of the ‘anti-inflammatory reflex’ [57]. Furthermore, it has been shown that the efferent arm of the ‘anti-inflammatory reflex’ to lipopolysaccharide challenge is primarily the splanchnic sympathetic nerve acting on immune cells in the spleen [58] (Figure 1).

Thus, there is no simple statement like ‘norepinephrine is anti- or pro-inflammatory’. It is better to say ‘norepinephrine modulates immune function in a context-dependent manner’. It gets even more complex when the release of co-transmitters, which is dependent on the firing rate of sympathetic nerve fibers [59], and neuroanatomical facts are taken into account, because all known co-transmitters like NPY, ATP, and nitric oxide are potent immune modulators and, thus, effects superimpose on each other. To answer the question about the role of the SNS in inflammation, research at the single cell level is important to understand basic regulatory mechanisms. However, the complexity of the interrelation between different factors is challenging. In addition, it has to be respected that the SNS also interacts with non-immune cells to modulate release of inflammatory mediators. For example, endothelial cells can be stimulated to increase release of IL-6 via NE and ATP from SNS nerve terminals [60].

Another approach to understand the role of the SNS in inflammation is to investigate the overall effect of SNS activity on clinical outcomes. Well-known clinical phenomena, like the reactivation or first occurrence of chronic inflammatory disorders like colitis or asthma during or after episodes of psychological stress, have been directly linked to the activation of the autonomic nervous system [61,62]. Influence of the SNS on inflammation at a systemic level has been demonstrated for several disease models and entities like sepsis [17], colitis [63], allergic asthma [47,61], chronic eye inflammation [64], arthritis [51,65], endometriosis [66], T helper type 1-mediated skin diseases [67], influenza A [68], Chagas disease [69], and chronic regional pain syndrome [70].

Evidence has also accumulated to show that chronic activation of the SNS by changing function of immune cells contributes to hypertrophy and fibrosis of the heart [71]. Similarly, in a mouse model of primary biliary cirrhosis, blockade of sympathetic activity improved fibrosis [72]. It has been shown in a restraint stress paradigm influenza model that the sympathetic component of the stress response, possibly due to limiting otherwise detrimental specific effector cell activation, together with glucocorticoids are responsible for better survival after experimental infection [73].

There is also evidence that different forms of cancer might be influenced by the SNS, including from experimental animal data, epidemiological studies that show the use of beta-blockers is beneficial for breast cancer and melanoma, and studies showing that psychological stress might play a role in the pathogenesis of some cancers [74]. Taken together, these studies show that the SNS plays an important role in several immune-mediated or immune-related diseases.

Clinical models demonstrate that influencing the sympathetic response impacts on the outcome. In a model of acute septic inflammation, the adrenergic system has a profound influence on cell proliferation, apoptosis, and circulating immune cell subpopulations [75]. In a model of polymicrobial sepsis by cecal ligation and puncture, mechanisms through α-ARs increase mortality. In the same system, it has been described that tyrosine hydroxylase (TH) is markedly increased in sympathetic fibers of the small intestine-associated SNS, resulting in enhanced NE release [76]. Therefore, not only is response of immune cells to SNS stimuli highly context-dependent, but the nervous system itself also underlies plasticity depending on the inflammatory context.

From our point of view, arthritis is the best investigated disease entity concerning the influence of the SNS on the inflammatory process. Therefore, the next section focuses on this chronic disease to introduce current concepts of SNS influence on inflammation.

## The sympathetic nervous system and arthritis

Sympathectomy in patients with rheumatoid arthritis was reported as early as 1927 (mentioned in [77]), followed by several reports showing that pain as well as joint swelling improved upon sympathectomy (for example, [77]). In a double blind study in 1986, however, overall pain decreased but no changes were recorded with respect to morning stiffness or joint tenderness [78]. This is in contrast to reports in animal models that sympathectomy leads to less severe disease - for example, in carrageenan-induced arthritis [79] or adjuvant arthritis in rats [80]. In the latter model, spontaneous hypertensive rats, which show higher activity of the SNS, developed more severe arthritis [81]. It seems that this proinflammatory effect of the SNS on early adjuvant arthritis is caused by an increase in T helper type 1 lymphocyte (Th1) and Th17 responses [82].

**Figure 3:**
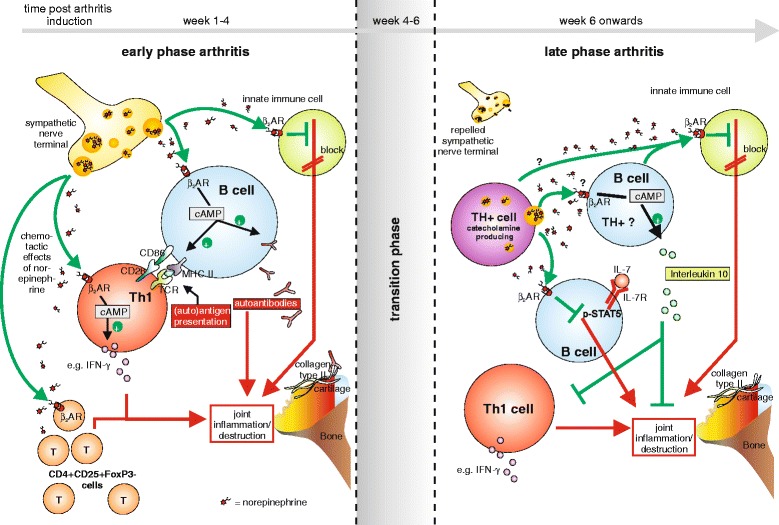
**Current model of sympathetic nervous system influence in arthritis.** In early arthritis (left panel), the sympathetic nervous system (SNS) supports inflammation in the joint through a proinflammatory influence on adaptive immune cells; for example, increased specific antibody production by B cells and increased proinflammatory activity of T cells. The SNS also inhibits innate immune cells via stimulation of β2 adrenoceptors (β_2_ARs), although the net outcome of SNS influence in the early phase is proinflammatory. Then, during the transition phase, we hypothesize that the influence of the SNS changes from pro- to anti-inflammatory. In the later stages, central regulation of the inflammatory process is less important, since sympathetic nerve fibers are repelled from the inflamed area and secondary lymphoid organs. However, local sympathetic influence becomes increasingly important, indicated by the appearance of catecholamine-producing, tyrosine hydroxylase-positive (TH+) cells, which have a dominant anti-inflammatory effect. Possible mechanisms of action are paracrine and autocrine in manner; for example, inhibiting proinflammatory interleukin (IL)-7 receptor-positive B cells, increasing the activity of IL-10-producing anti-inflammatory B cells, or inhibiting innate immune cells via β_2_AR-mediated effects. AR, adrenoceptor; cAMP, cyclic adenosine monophosphate; CD, cluster of differentiation; FoxP3, forkhead box P3; IFN, interferon; MHC, major histocompatibility complex; pSTAT5, phosphorylated-signal transducer and activator of transcription 5; TCR, T-cell receptor; Th1, T helper 1 cell.

A proinflammatory activity of the SNS was also shown in the collagen type II model of arthritis [51]. In this model, proinflammatory CD4 + CD25 + FOXP3- cells induced this effect [83] (Figure 3). These results in human and animal studies seem to be contradictory. However, these divergent results can be explained by the importance of the time point of sympathetic intervention. This was clearly shown in the collagen type II model of arthritis in DBA/1 mice, where early sympathectomy leads to less severe disease, but late sympathectomy in the chronic phase of the disease clearly has the opposite effect, resulting in increased disease activity [51]. How can one explain this dichotomy?

It has long been known that innervation, which is usually dense in synovial tissue, is lost during experimental inflammation and in chronic inflammatory conditions [84]. However, more recent studies showed that the loss of innervation is a specific process and affects mainly sympathetic nerves fibers, whereas sensory nerves remain in the inflamed region [85], an observation reproducible in many inflammatory conditions of humans and rodents. Recent research demonstrates an active process possibly involving specific nerve repellent factors [86].

**Figure 4:**
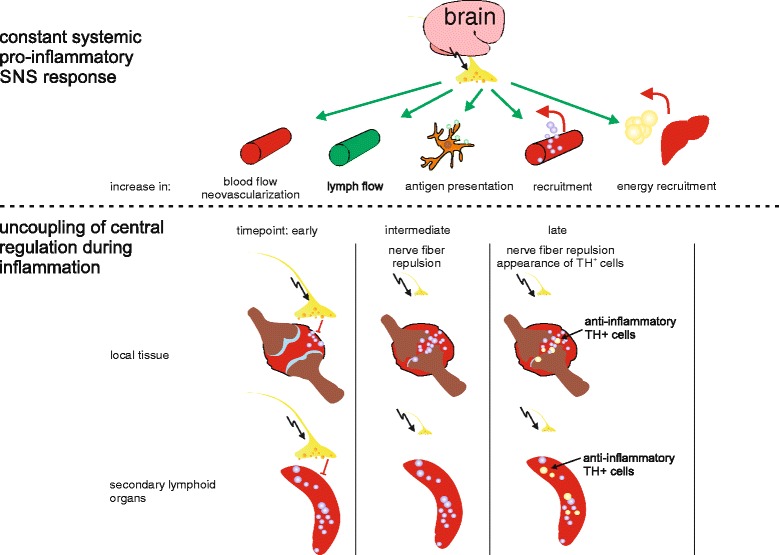
**Morphologic adaptation to persistent inflammation.** Centrally controlled increase of sympathetic nervous system (SNS) activity is a basic response to inflammation. The constant increase in SNS activity supports inflammation in several ways; for example, increasing blood flow, lymph flow, antigen presentation, and liberation of energy-rich fuels like lipids and glucose from adipose tissue and liver. However, the specific interaction with immune cells in secondary lymphoid organs and at local sites of inflammation (for example, joints) shows a net anti-inflammatory effect. Therefore, to mount an effective immune response, non-specific support of inflammation on a systemic level is maintained, while the anti-inflammatory influence on a local level is decreased and uncoupled from central regulation through repulsion of sympathetic nerve fibers and the appearance of tyrosine hydroxylase (TH) + catecholamine-producing cells during the inflammatory process. In the end, a systemic proinflammatory configuration is established, which helps to optimally clear the antigen. However, if inflammation persists, like during chronic inflammation, this constant increase in SNS activity and resultant catabolic state is detrimental to the body and results in known disease sequelae of chronic inflammatory conditions, like cachexia, diabetes, hyperlipidemia, high blood pressure, increased cardiovascular risk, and so on.

As a compensatory mechanism for this deprivation of sympathetic neurotransmitters in the joint, cells that are capable of producing neurotransmitters accumulate [87]. These TH-positive catecholamine-producing cells modulate inflammation dependent on the model used. In a model of lung injury, α2-dependent proinflammatory effects of catecholamine-producing phagocytes were postulated [88]. On the other hand, in multiple sclerosis [89] and human and experimental arthritis [87,90,91], catecholamine-producing cells have anti-inflammatory potential. These TH-positive cells are sensitive to sympathectomy with 6-hydroxydopamine (a neurotoxin) or anti-dopamine beta hydroxylase antibodies [90]. Since TH-positive cells dominate the later phase of collagen type II-induced arthritis in the joint (they are also present in synovial inflammation in chronic rheumatoid arthritis), it is not surprising that depletion of these cells by sympathectomy leads to aggravation of arthritis in the late phase [51]. At the moment, however, the mechanism of anti-inflammatory action has not been fully established in arthritis. Possibly, cAMP content in TH-positive cells is increased by autocrine mechanisms. In this respect, it has been shown for regulatory T cells (Tregs) that cAMP can be used as a direct immunosuppressive agent by transferring cAMP molecules from Tregs via gap junctions into target cells [92]. Due to high concentrations of neurotransmitters in the vicinity of TH-positive cells, however, stimulation of β2-ARs on innate immune cells might be the dominant immunosuppressive mechanism (Table 1, Figures 3 and 4).

An influence on adaptive immune cells like B cells has also been shown. In the collagen-induced arthritis model, B cells expressing IL-7 receptor are proinflammatory [46]. However, stimulation of β2-AR on B cells results in loss of proinflammatory activity by inhibiting IL-7 receptor downstream signaling (Figure 3). Another possible explanation for the anti-inflammatory effects of TH-positive cells is increased anti-inflammatory function, which is augmented by catecholamines in an autocrine or paracrine manner via ARs. In collagen type II-induced arthritis, it has been shown that a subpopulation of B cells might play a role in this respect [45]. NE via β2-AR increased IL-10 production from B cells from arthritic animals (Figure 3), and these cells were anti-inflammatory when re-injected into arthritic animals [45]. One might speculate that these B cells, which can be TH-positive, are stimulated by catecholamines produced by TH-positive cells in the joint in an autocrine/paracrine manner (Figure 3).

## The purpose of activating the sympathetic nervous system in inflammation - exemplified by synovial inflammation

So far, we introduced a new model of neuroimmune regulation specified in arthritis. All these elaborate mechanistic and structural adaptations during inflammation need to serve some purpose, however, otherwise they would not have been positively selected during evolution. In recent hypothetical modeling, a framework was developed that tries to explain the underlying meaning.

An activated immune system needs a significant amount of energy above that required for the normal non-inflamed state [93]. The activation of the SNS and the HPA axis at the beginning of inflammation helps to provide enough energy, because activation of these axes mobilizes energy-rich fuels mainly by increasing lipolysis, glycogenolysis, muscle protein breakdown, and gluconeogenesis (Figure 4). At the beginning of an inflammatory innate immune response, the SNS but also HPA axis support inflammation by non-specific means; for example, mobilization of leukocytes [22,26], increasing blood pressure and heart rate, increasing lymph flow [21], plasma extravasation [94], antigen uptake and presentation [37] (Figure 4). In this initial phase of inflammation, SNS activity also ‘programs’ adaptive immune cells via β2-AR - for example, B cells to produce increased amounts of antibodies and T cells to produce more or less cytokines dependent on the context of activation [95]. This mainly proinflammatory action takes place on a systemic level in secondary lymphoid organs like the spleen and lymph nodes, where immune cells are programmed and then released to attack the intruder.

At the local site of inflammation, however, SNS activity contributes primarily to anti-inflammatory mechanisms, mainly by direct influence of neurotransmitters on immune cells [4]. Besides the local promotion of regulatory B cells (see above), also macrophages stimulated via the β2-ARs acquire an anti-inflammatory M2 phenotype [96] and β2-AR stimulation also inhibits TNF production [97] (Figure 2). On the other hand, stimuli via α-ARs are proinflammatory: for example, α2-AR stimulation increases reactive oxygen species in macrophages [98]. Therefore, the net outcome of stimulating ARs on immune cells strongly depends on the receptor engaged and, therefore, on the receptor expression pattern (which might change during the course of inflammation [4,45]) and neurotransmitter concentration, because NE binds preferentially to α-ARs, only binding to β-ARs at high concentrations (for example, [99]). However, why do some immune cells, like macrophages, express both α-ARs and β-ARs, which will counteract each other in terms of immunoregulation? One possible explanation is that, due to the different binding affinities of NE to these AR subtypes, this system can be used as a distance detector to the source of catecholamines.

In this respect, repulsion of sympathetic nerve fibers from inflamed tissue makes sense, since it is not favorable to inhibit the immune response (high concentrations of catecholamines preferentially stimulate anti-inflammatory β-ARs) before the antigen is cleared (Figure 4). Therefore, this distance detector system (simultaneous expression of α-AR and β-AR on immune cells) provides a means for the body to define sites of permitted inflammation (low SNS fiber density, low catecholamine concentration) and, on the other hand, prevent uncontrolled spreading of inflammation by preventing bystander activation (high SNS fiber density, high catecholamine concentration) (Figure 2).

To get an impression of the contribution of SNS to local anti-inflammatory mechanisms, the eye is a good example. The eye is known as an exceptional immune-privileged site, dominated by anti-inflammatory mechanisms. It has been shown that sympathetic denervation of the eye leads to a decrease in anti-inflammatory molecules, like tumor growth factor-β, which results in a complete loss of the immune-privileged status [100]. Therefore, repulsion of SNS fibers from inflamed tissue is an effective means to increase local inflammation (Figures 2 and 4). This has been positively selected during evolution to clear invading microbes but not to serve chronic autoimmune inflammation.

We hypothesize that catecholamine-producing cells start to play a role in the later inflammatory phase, possibly as a compensatory mechanism for the local loss of SNS fibers. These TH-positive cells can be anti-inflammatory as described above. One might argue that it is easier to just shut down SNS activity at the systemic level than to repel nerve fibers from local inflamed tissue, but SNS activity stays high during many chronic inflammatory conditions (for example, [101]). Concerning the energetic aspect discussed above, this is beneficial in terms of providing enough energy to feed the activated immune system on a systemic level. In contrast to the SNS activity, which is still high in chronic inflammation, HPA axis activity is relatively reduced, not down to normal, but to a level without immunosuppression, to not disturb the local immune response (Figures 3 and 4).

Overall, the system takes on an ‘inflammation configuration’, including repulsion of sympathetic nerve fibers from local inflamed tissue to create an area of permitted inflammation, high SNS activity on a systemic level, and reduced HPA activity without local immunosuppression, but provision of energy-rich fuels is still maintained and important (Figure 4).

These processes are positively selected during evolution to serve short-term acute inflammation [93,102]. If these processes persist for too long, they cause harm because the body is in a constant state of catabolism and volume overload. Known disease sequelae in chronic inflammatory conditions can be explained by this constant activation of the SNS and HPA axis and the resultant catabolic state, like cachexia, high blood pressure, insulin resistance, and so on [93,102].

## Potential clinical and therapeutic implications for chronic inflammatory processes

From the current conceptual and experimental knowledge, certain hypotheses can be derived about potential clinical and therapeutic approaches that might improve clinical practice. Clinical data applying the current knowledge specifically on sympathetic regulation of inflammation is scarce. However, one promising approach that underscores the importance of sympathetic downstream signaling in anti-inflammation is the inhibition of phosphodiesterase (PDE)4, an enzyme that degrades cAMP. Increasing cAMP by inhibiting this enzyme shows promising results in psoriatic arthritis, which led to the approval of the PDE inhibitor apremilast for this disease entity [103]. PDE inhibitors are also currently being tested for several other clinical entities; for example, psoriasis, rheumatoid arthritis, and Behcet’s syndrome [103]. Taking into consideration that a general increase in cAMP might also support detrimental effects as discussed above, it is noteworthy that PDE4 is the predominant PDE isoform expressed in immune cells [104]. However, whether increasing cAMP by pharmacologic PDE inhibition will support disease sequelae is not clear at the moment and further research is needed. Right now, neuroimmunology in the sense presented in this review is on the verge of clinical translation. In terms of sympathetic control of inflammatory arthritis a possible approach is to follow the success seen in animal models and put effort into developing novel cellular therapies; for example, after induction of TH in certain immune cells or treatment of B cells with sympathetic stimuli to increase their regulatory potential. On the other hand, the systemic permanent overactivation of the SNS as discussed above could also be a potential target for intervention; for example, by psychological or pharmacological means. However, clinical data are missing at the moment and further research is warranted. For this research an approach to support local activation of sympathetic mechanisms, like increasing cAMP in immune cells (for example, PDE4 inhibition) but on the other hand decreasing systemic SNS activation to prevent disease sequelae, needs to be the focus.

## Conclusion

Inflammation causes increased activity of the SNS with release of NE and co-transmitters in lymphoid organs and inflamed local sites. Immune cells carry receptors (for example, ARs) to detect and process signals from the SNS. The reaction of the immune cell to neurotransmitters is variable depending on the context of receptor engagement (activation state of the cell, expression pattern of neurotransmitter receptors, microenvironment, cytokine milieu, and distance from the catecholamine source (concentration)).

On a systemic level, the signals from the SNS are proinflammatory in the initial phase of inflammation, whereas anti-inflammatory effects are dominant in the late or chronic phases of an inflammatory response, at least in collagen-induced arthritis. Upon initiating an inflammatory process, the body adopts an ‘inflammatory configuration’ with increased systemic SNS and HPA axis activity. This reaction can be interpreted as an ‘energy appeal reaction’ resulting in the provision of enough energy-rich fuels, like glucose and free fatty acids, to fulfill the needs of an activated immune system.

If inflammation becomes chronic, as in chronic inflammatory illness, the system changes into a ‘chronic inflammatory condition’ that is characterized by 1) still increased systemic activity of the SNS, 2) still increased activity of the HPA axis but without immunosuppression (glucocorticoid receptor desensitization and inadequacy), and 3) local repulsion of SNS fibers from inflamed tissue, including lymphoid organs, to create zones of permitted inflammation. The immune response is more or less uncoupled from central regulation to avoid the anti-inflammatory influence of the brain. All mechanisms ensure an optimal fight against an antigen.

These adaptations are evolutionarily positively selected to clear the antigen, usually an intruding microbe. However, if a ‘chronic inflammatory configuration’ persists, as in autoimmunity, the effects are detrimental because of the persistently increased SNS activity, HPA activity, and the resultant chronic catabolic state. This leads to known comorbidities in chronic inflammatory disease, like cachexia, high blood pressure, insulin resistance, and increased cardiovascular mortality. The challenge is now to translate this conceptual knowledge into clinical benefit.

## Abbreviations

AR: Adrenoceptor
DTH: Delayed type hypersensitivity
HPA: Hypothalamic-pituitary-adrenal
IL: Interleukin
nACh: Nicotinic acetylcholine
NE: Norepinephrine
NPY: Neuropeptide Y
PDE: Phosphodiesterase
SNS: Sympathetic nervous system
TH: Tyrosine hydroxylase
TNF: Tumor necrosis factor
Treg: Regulatory T cell
VNS: Vagal nervous system
